# Gender Differences Associated with Hyper-Inflammatory Conditions in COVID-19 Patients

**DOI:** 10.14336/AD.2022.0830

**Published:** 2023-04-01

**Authors:** Fouzia Shoeb, Farzana Mahdi, Imran Hussain

**Affiliations:** ^1^Departments of Allied and Paramedical Sciences, and; ^2^Personalized and Molecular Medicine, Era's Lucknow Medical College and Hospital, ERA University, Lucknow, UP, India

**Keywords:** COVID-19, gender disparity, hyperinflammatory conditions, hyperferritinaemia, hematological dysfunctions, cytokinaemia, coagulopathy, liver inflammation

## Abstract

COVID-19 has been associated with various hyper-inflammatory conditions (HICs) such as macrophage activation, hematological dysfunction, cytokinaemia, coagulopathy, and liver inflammation. However, it is not clear if the differences in the disease severity and mortality shown by male and female COVID-19 patients are associated with these HICs. Here, we review the literature and present supporting laboratory data on the gender differences associated with various HICs in COVID-19 patients. We measured plasma/serum levels of various HIC specific clinical markers in severe male (N=132) and severe female (N=78) COVID-19 patients. The result revealed that all clinical markers were highly elevated above the normal in both male and female COVID-19 patients. However, a comparison of AUROC (area under the receiving operative characteristics) of specific clinical markers revealed that elevation in serum ferritin (marker for macrophage activation), and neutrophil to lymphocyte (N/L) ration (marker for hematological dysfunction) was much higher in male compared to the female COVD-19 patients. Further, univariate regression analyses revealed that male COVID-19 patients had two times higher risks than female patients for developing macrophage activation (OR 2.36, P=0.004)), hematological dysfunctions (OR 2.23, P=0.01), coagulopathy (OR 2.10, P=0.01), and cytokinaemia (OR 2.31, P=0.01). Similar results were obtained in bivariate analyses. Survival curve analysis showed that male COVID-19 patients had relatively short survival duration than female COVID-19 patients (hazard ratio 2.0, 95% CI 1.3-3.7, P=0.01). The above findings suggest that the high mortality rate in male COVID-19 patients compared to the female could be due to higher prevalence and severity of various HICs.

COVID-19 (Coronavirus diseases-2019) is a highly contagious disease caused by a novel strain of coronavirus classified as severe acute respiratory syndrome corona virus 2 (SARS-CoV-2) [1]. Several inflammatory conditions have been linked with COVID-19, including activation of macrophages, hematological dysfunctions, and cytokinaemia or cytokine storm, defined by upregulation of C-reactive protein (CRP), interleukins (IL), and tumor necrosis factor-alpha (TNF-α) [2-4]. Further, coagulopathy, as identified by increased D-dimer formation, has been reported as an independent risk factor for mortality in COVID-19 patients [5]. Liver inflammation caused by SARS-CoV-2 has also been linked to an increased risk of death in COVID-19 patients [6-8). Evidence suggests that SARS-related acute respiratory distress syndrome was also caused by excessive activation of inflammatory cytokines (TNF-α, IL-6, IL-8, and IL-10) and acute inflammatory proteins. Impaired organ function and high death rate were also directly related to increased immunological response, including high levels of the proinflammatory cytokine IL-6 in Middle East Respiratory Syndrome (MERS) [9].

Epidemiological data suggests that COVID-19 is mounting high mortalities in older people, specially, those of age 50 years or higher [10,11]. The risk of mortality increases further in patients with pre-existing co-morbidities such as hypertension, diabetes, respiratory complications, and others [5-8,12]. Furthermore, several lines of evidence suggest that males carry significantly higher risk for COVID-19 associated mortality than females of the same age [12] While the mechanisms associated with these differences are not clear, findings such mechanisms or risk factors may help in developing appropriate preventing therapies, and also in designing gender specific personalized treatments for post COVID-19 recovery phase.

Accumulating evidence suggests that development of various hyperinflammatory conditions (HICs) greatly increases the risk for mortality in COVID-19 patients [3,4,13-16]. These HICs have been used recently for defining hyperinflammatory syndrome and predicting the risk of severity and mortality in COVID-19 patients [17]. However, it is not clear whether these HICs contribute to gender differences associated with COVID-19 severity and mortality. In this commentary article, while we reviewed literature on various HICs in COVID-19 patients, preliminary laboratory data on gender differences in various HICs and associated clinical markers including, macrophage activation (serum ferritin), hematological dysfunction (neutrophil to lymphocyte ratio - NLR), coagulopathy (D-dimer), cytokinaemia (C-reactive protein: CRP), and liver inflammation (aspartate aminotransferase: AST) are also presented. We also present the impact of pre-existing co-morbidities on the severity and mortality associated with COVID-19.

The study was approved by the Institutional Ethics Committee of Era’s Lucknow Medical College and Hospital, ERA University, Lucknow, India (Ref. No.: ELMC & H/R-Cell/EC/2020/272). All procedures were carried out in compliance with the ethical requirements of ELMC&H, Era University. A total of 132 male and 78 female severe COVID-19 patients were included in this study. COVID-19 was diagnosed using body temperature and the RT-PCR method. The exclusion criteria were patients with pregnancy, known malignancies and mental disabilities. A self-administered questionnaire was used to collect individual demographic data, clinical history of diabetes, hypertension and renal disease, as well as other clinical data obtained from hospital records under the supervision of an expert clinician. After obtaining informed consent from each patient, 2 mL of blood was collected in tubes containing ethylene diamine tetra acetic acid (EDTA). In addition to severe COVID-19 patients mentioned above, a group of mild COVID-19 patients, comprising of 24 males and 13 females, was also included for comparing the elevation in the serum levels of various clinical markers. According to the Indian Council of Medical Research (ICMR), New Delhi, India, patients with a respiratory rate of less than 24 breaths per minute and a SpO2 of greater than 94 percent on room air were classified as mild, while patients with a respiratory rate of more than 30 breaths per minute or a SpO2 of less than 90 percent on room air were classified as severe. All the laboratory measurements were performed on a daily basis using fresh blood samples and following the instructions provided by the manufacturer using appropriate calibrators and controls (see supplementary data). Statistical analyses were performed using SPSS and Prism (GraphPad) software. Student's *t* test and ANOVA were used to find differences between the groups for various clinical markers. Normality test analysis was used to find the distribution pattern of the data, and whenever data failed normality test, unpaired non-parametric *t*-test or Mann Whitney test were applied to find the significance. Pearson product moment correlation analysis was performed to find the relationship among various laboratory markers. Univariate, multivariate and binary logistic regression analyses were performed using a 2 x 2 contingency table and applying Fischer's exact test. Odds ratio (OR) and sensitivity at 95% confidence intervals (CI) were calculated to find the association of various risk factors and HICs with the severity and mortality in COVID-19 patients. Survival curves were drawn and compared using Kaplan and Meier method and the hazard ratio was calculated using logrank (Mantel-Cox) test. A P value ≤ 0.05 was considered significant.

We first evaluated the association of various risk factors/comorbidities with the severity and mortality in severe male and female COVID-19 patients and the result are presented in the Table 1. Since the origin of COVID-19, several studies have shown that individuals with premorbid conditions carry significantly higher risk for developing COVID-19 [18-23]. For instance, present of diabetes [18], kidney disease [19], hypertension [20], cardiovascular disease [21], dyslipidemia [22], and mental disorders [23] have been found to increase the severity and risk for mortality in COVID-19 patients. Intriguingly, while COVID-19 develops mainly in older people, we observed that a high percentage (20%) of young males (age ≤ 45 years) also developed severe COVID-19 compared to the young females (5%) (Table 1). Further, univariate regression analyses revealed that old age (OR 3.41, P= 0.0137), hypertension (OR 3.38, P=0.0037) were strongly associated with severe male COVID-19 patients compared to the female patients. On the other hand, diabetes (OR 0.49, P=0.0210) was strongly associated with severe female COVID-19 patients. In the binary analysis, diabetes+ hypertension (OR 2.76, P=0.0510, Table 1) and renal disease + hypertension (OR 3.70, P=0.0162, Table 1) were strongly associated with severe male COVID-19 patients compared to the female COVID-19 patients. These findings are in agreement with various reports published earlier [18-23].

**Table 1:** Association of various risk factors (comorbidities) with male and female COVID-19 patients (Univariate analyses).

Risk factors	Male N=132	Female N=78	Univariate OR (95% CI)	*P*	Sensitivity (95 %CI)
Age (years) ≤ 45 (n, %) ≥ 46 (n, %)	025 (18.94) 107 (81.06)	05 (0.6.41) 73 (93.50)	1 (Ref) 3.41 (1.25 - 9.32)	0.01	0.83 (0.65 - 0.94)
Marital status Married (n, %) Unmarried (n, %)	110 (83.33) 022 (16.67)	68 (87.18) 10 (12.82)	1 (Ref.) 0.74 (0.33-1.65)	0.55	0.62 (0.54- 0.69)
Diabetes Yes (n, %) No (n, %)	32 (24.24) 100 (75.76)	31 (39.74) 47 (60.26)	1 (Ref.) 0.49 (0.27-0.89)	0.02	0.51 (0.38-0.36)
Renal Disease Yes (n, %) No (n, %)	25 (19.94) 107 (81.06)	15 (19.24) 63 (80.76)	1 (Ref.) 1.07 (0.52-2.20)	1.00	0.64 (0.47-0.79)
Hypertension Yes (n, %) No (n, %)	33 (25.00) 99 (75.00)	07 (8.97) 71 (91.03)	1 (Ref) 3.38 (1.42-8.10)	0.004	0.83 (0.67-0.93)
Diabetes + renal disease Yes (n, %) No (n, %)	19 (14.39) 123 (59.09)	12 (15.38) 66 (84.61)	1 (Ref) 2.31 (1.23-4.37)	0.89	0.75 (0.63-0.85)
Diabetes + Hypertension Yes (n, %) No (n, %)	21 (15.91) 111 (84.09)	05 (06.41) 73 (93.59)	1 (Ref) 2.76 (1.00-7.65)	0.05	0.81 (0.61-0.93)
Renal disease + Hypertension Yes (n, %) No (n, %)	22 (16.67) 110 (83.33)	04 (05.13) 74 (94.87)	1 (Ref) 3.70 (1.20-11.0)	0.02	0.85 (0.65-0.96)

OR: odds ratio, CI: confidence interval, N: total number of patients, (n, %): the number, and percentage of patients with (Yes) or without (No) specified conditions.

We next performed serum analyses of various clinical markers associated with specific hyperinflammatory conditions (HICs) and the results are shown in Table 2. For gender differences, we performed statistical analyses in male and female COVID-19 patients separately. We have also included control/reference values provided by the respective manufacturer form validation of the results. These control values were verified in the lab and found to be comparable. In addition, we also included a group of males (N=24), and female (N=13) patients with mild COVID-19 symptoms for comparing elevation in various clinical markers with that of severe COVID-19 patients. As shown in the table 2, the serum levels of all clinical markers were sharply elevated in severe COVID-19 patients compared to the controls (Reference values) and mild COVID-19 patients. A comparison between male and female COVID-19 patients by AUROC analyses suggests that while serum ferritin and NLR were increased in all COVID-19 patients, increase was significantly more pronounced in male compared to the female COVID-19 patients. Likewise, D-dimer, CRP, or AST were also sharply increased in all patients, however, male to female differences in AUROC were not significant (Table 2). Figure 1 shows the dot plots of various clinical markers in severe male (blue) and female (pink) COVID-19 patients. The elevation in serum ferritin and NLR ration was significantly mush higher in male compared to the female COVID-19 patients. These findings are in agreement with several previously published studies, which have reported significant elevation in various clinical markers including ferritin and NLR in severe COVID-19 patients [24-26].

Using the serum values of various clinical markers measured above, we defined various HICs in the same way as described and applied recently by Webb et al for defining various criteria of hyperinflammatory syndrome (HIS) in severe COVID-9 patients [17]. In this context, a serum ferritin value ≥700 μg/L was used for defining macrophage activation, a neutrophil to lymphocyte ratio (NLR) ≥10 for hematological dysfunction, a D-dimer value ≥1.5 μg/mL for coagulopathy, a CRP value ≥15 mg/dL for cytokinaemia, and an AST value ≥ 100 U/L was used for predicting liver inflammation [17]. Based on these cut-off values, we analyzed the prevalence (as %) and association strength (Odds ratio: OR) of various HICs with severe male (132) and female (78) COVID-19 patients and the results are shown in the Table 3 and 4, and Supplementary Table 1 and 2. For measuring association strength, we used univariate, multi-variate and binary regression methods.

**Figure 1.**
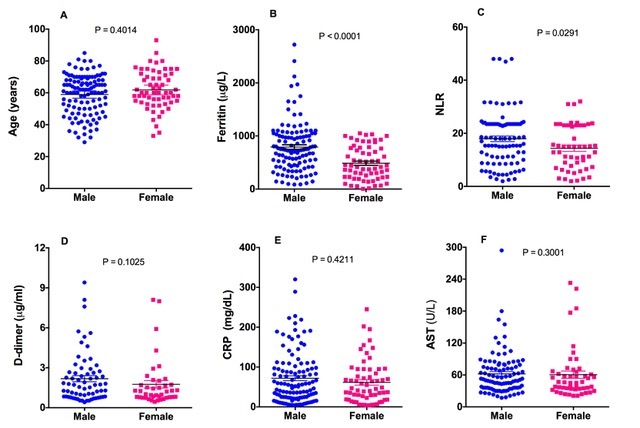
Dot plot of plasma/serum values of various clinical markers in severe male (blue dots) and severe female (pink dots) COVID-19 Patients. (A) Age, (B) serum ferritin, (C) NLR, (D) D-dimer, (E) CRP and (F) AST. Significance was calculated using unpaired non-parametric Mann-Whitney *t*-test. Error bars represent SE. A *P* value < 0.05 was considered significant.

Table 3 shows univariate analyses of the association of various HICs with severe male and female COVID-19 patients. As can be seen from the table 3, macrophage activation was highly prevalent and strongly associated with severe male COVID-19 patients compared to the female COVID-19 patients. The calculated OR 2.36 (P=0.0041) suggests that the risk of male COVID-19 patients had for developing macrophage activation was two times the risk of female COVID-19 patients. In addition, as shown in table 2, AUROC of ferritin was also significantly high in male COVID-19 patients (P <0.0001) compared to the female COVID-19 patients. Together, these findings suggest that SARS-CoV-2 infection leads to more severe inflammatory reactions in male compared to the female COVID-19 patients, which is in agreement with several reports published earlier [24-30]. Similarly, NLR ≥10, which has been used as a good indicator for hematological dysfunctions in many inflammatory diseases including inflammatory bowel disease, type-2 diabetes, thyroiditis, irritable bowel disease and COVID-19 [31-35], was also highly prevalent in male (74%) compared to the female (54%) COVID-19 patients. The calculated OR was 2.1 (95% CI 1.0-4.2, P=0.0095), which suggest that the risk of male COVID-19 patients had for developing hematological dysfunctions was two times the risk female patients. Lymphocytopenia (reduced lymphocyte count) appeared to be the most prominent feature associated with the increased severity of hematological dysfunctions in male COVID-19 patients. Approximately 86% male compared to only 64% female COVID-19 patients developed lymphocytopenia suggesting that SARS-CoV-2 infection induces more severe hematological dysfunction in male compared to the female COVID-19 patients. These findings are in line with several other studies, which have reported severe lymphocytopenia in patients infected with SARS-CoV-2 or other viruses [3,13,35-40]. Coagulopathy and cytokinaemia were also more prevalent and strongly associated with severe male COVID-19 patients compared to the severe female COVID-19 patients. As shown in the Table 3, 61% male compared to 42% female COVID-19 patients developed coagulopathy, and the calculated OR was 2.10 (95% CI 1.20-3.70, P=0.0146), suggesting that male COVID-19 patients had two times more risk for developing coagulopathy than the female COVID-19 patients (Table 3). However, AUROC analysis of serum D-dimer did not reveal any significant difference between male and female COVID-19 patients (Table 2). Coagulopathy has been suggested as an independent risk factor for the mortality in COVID-19 patients in several investigations [5,41-44]. Regarding cytokinaemia, 41% male compared to only 25% female COVID-19 patients developed cytokinaemia, and the calculated OR was 2.3 (95% CI 0.65- 2.7, P=0.0104), suggesting that male COVID- 19 patients also had two times more risk for developing cytokinaemia than the female COVID-19 patients. These findings support various studies, which have shown a sharp increase in several inflammatory cytokines and chemokines including CRP in severe COVID-19 patients [2-4, 13-16, 25].

**Table 2:** Serum values (range*) of various laboratory/clinical markers in severe COVID-19 patients.

Clinical markers	Male	Female	AUROC (95% CI)	P
Ferritin (mg/L) Ref.** Mild Severe	18-464 84-790 57-2720	06-262 05-523 42-1050	0.63 (0.46-0.80) 0.73 (0.64-0.83)	0.142 0.000
NLR Ref. Mild Severe	01-03 03-15 02-38	01-03 02-13 03-33	0.57 (0.39-0.72) 0.67 (0.57-0.77)	0.09 0.01
D-Dimer (mg/ml) Ref. Mild Severe	0-0.5 0.41-2.7 0.43-9.4	0-0.5 0.55-2.4 0.45-8.1	054 (0.35-0.74) 0.61 (0.43-0.79)	0.68 0.29
CRP (mg/dL) Ref. Mild Severe	0-10 05-145 04-320	0-10 05-120 03-245	0.52 (0.34-0.70) 0.57 (0.45-0.69)	0.85 0.21
AST (U/L) Ref. Mild Severe	17-59 16-89 18-294	14-36 19-75 18-922	0.55 (0.53-0.71 0.63 (0.51-0.73)	0.64 0.07

* Serum values of clinical markers are given as minimum-maximum range,

** Reference (Ref.) values as provided by the respective manufacturers were verified in the lab, which were found to be comparable. AUROC - Area under the receiver operating characteristics NLR - Neutrophil to lymphocyte ratio CRP - C-reactive protein AST - Aspartate aminotransferase

In addition to the above HICs, liver/hepatic inflammation has been consistently reported in patients with COVID-19 [45-50]. Intriguingly, in contrast to the macrophage activation, hematological dysfunction, coagulopathy and cytokinaemia, which were strongly associated with severe male COVID-19 patients, we did not find significant gender differences in the prevalence of liver/hepatic inflammation in COVID-19 patients. As shown in Table 3, 11% male compared to 13% female COVID-19 patients developed liver/hepatic inflammation. The calculated OR was 0.81 (95% CI 0.34-1.90), which suggests that female COVID-19 patients had slightly higher risk for developing liver inflammation than the male COVID-19 patients, however, the risk wasn’t' significant (P=0.6574). The average value and the AUROC of AST, respectively, were higher in female compared to the male COVID-19 patients but significance couldn't be established (P > 0.05, Table 2). In conclusion, the data discussed above based on the univariate logistic analyses suggest that macrophage activation, hematological dysfunction, coagulopathy and cytokine-aemia were predominantly and strongly associated with severe male COVID-19 patients, whereas liver inflammation may be slightly more prevalent in female COVID-19 patients.

**Table 3:** Association of various HICs with male and female COVID-19 patients (Univariate analyses).

HICs ↓	Male N=132	Female N=78	Univariate OR (95% CI)	*P*	Sensitivity (95 %CI)
Macrophage activation Positive (n, %) Negative (n, %)	82 (62.12) 50 (37.88)	32 (41.03) 46 (58.97)	1 (Ref) 2.36 (1.33-4.20)	0.004	0.72 (0.627- 0.80)
Hematological dysfunction Positive (n, %) Negative (n, %)	98 (74.24) 34 (25.76)	44 (56.41) 34 (43.59)	1 (Ref.) 2.23 (1.23-4.03)	0.01	0.69 (0.607-0.77)
Coagulopathy Positive (n, %) Negative (n, %)	80 (60.61) 52 (39.39)	33 (42.31) 45 (57.69)	1 (Ref.) 2.10 (1.2-3.7)	0.01	0.71 (0.610-0.79)
Cytokinaemia Positive (n, %) Negative (n, %)	54 (40.91) 78 (59.09)	18 (23.08) 60 (76.92)	1 (Ref.) 2.31 (1.23-4.37)	0.01	0.75 (0.634-0.85)
Liver inflammation Positive (n, %) Negative (n, %)	014 (10.61) 118 (89.39)	10 (12.82) 68 (87.18)	1 (Ref) 0.81 (0.34-1.90)	0.66	0.58 (0.37-0.780)

N: total number of patients, and (n, %): the number, and percentage of patients with (positive) or without (negative) specified conditions.

We also performed bivariate analyses to find the strength of association of any two HICs with severe male and female COVID-19. Evidence suggests that severe COVID-19 patients usually develop more than one HICs, with pre-existing comorbid conditions significantly increase the risk for mortality. Recently, Webb et al [17] have proposed that COVID-19 patients who develop two or more HICs may proceed to mechanical ventilation and death. We therefore performed categorical bivariate logistic regression analyses to find association strength of each pair of HICs with male and female COVID-19 patients. As shown in the supplementary Table 4, that macrophage activation + hematological dysfunction (39% vs. 21%, OR 2.50, P=0.0059), macrophage activation+ coagulopathy (42% vs. 23%, OR 2.53 P=0.0045), macrophage activation + cytokinaemia (15% vs. 5%, OR 3.30, P=0.043), and hematological dysfunction + coagulopathy (55% vs. 36%, OR 2.21, P=0.007) were more prevalent, and strongly associated with severe male COVID-19 patients compared to the female COVID-19 patients. These results support the outcome of univariate analyses as discussed above and suggest that male COVID-19 patients carry two times more risk for developing two HICs than females with severe COVID-19.

We next measured the impact of various HICs on the mortality in severe male and female COVID-19 patients. We separated male (N=34) and female (N=22) COVID-19 patients, who perished during the course of this study, and performed univariate and bivariate analyses considering all possible combinations of two HICs. The results are shown in Table 4 and Supplementary 6, respectively. Since the number of expired patients was low, we applied Fisher’s exact test to find the significance. In the univariate analyses (Table 4), coagulopathy (OR 7.20, P=0.0043) was strongly significantly associated with mortality in male COVID-19 patients compared to female COVID-19 patients. While proportion of patients displayed macrophage activation and cytokinaemia (Table 4) was also high in male COVID-19 patients, significance could not be achieved. Bivariate analyses presented in the Supplementary Table 2 revealed that hematological dysfunction + coagulopathy (OR 4.83, P= 0.0153) and coagulopathy+ cytokinaemia (OR 4.0, P=0.0448) were strongly associated with mortality in male COVID-19 patients compared to the female COVID-19 patients. While macrophage activation +hematological dysfunction (OR 2.22), macrophage activation + cytokinaemia (OR 3.00, Supplementary Table 2) and hematological dysfunction + cytokinaemia (OR 2.79) were also strongly associated with male COVID-19 patients, however, significance could not be achieved. Further, AUROC analyses of various clinical markers in these patients revealed significant (P= 0.022) differences in the level of serum ferritin, being higher in male compared to the female COVID-19 patients, whereas no significant differences were observed in the level of other clinical markers (data not shown). In conclusion, the data presented above suggest that a significantly high prevalence and severity of various HICs could be a reason for high mortality in male compared to the female patients with severe COVID-19. This was also reflected in the survival curve analysis or Kaplan-Meier analysis (Fig. 2) because the hazard male COVID-19 patients had for mortality was two times that of female COVID-19 patients (hazard ratio 2.0, 95% CI 1.3-3.7, P=0.006), and their in-hospital stay was also considerable short compared to the female COVID-19 patients.

**Table 4:** Association of various HICs with mortality in male and female COVID-19 patients (Univariate analyses).

HICs ↓	Male N=34	Female N=22	Univariate OR (95% CI)	*P*	Sensitivity (95 %CI)
Macrophage activation Positive (n) Negative (n)	21 13	12 10	1 (Ref) 1.30 (0.45-4.0)	0.78	0.62 (0.44- 0.78)
Hematological dysfunction Positive (n) Negative (n)	29 05	17 05	1 (Ref.) 1.71 (0.43-6.7)	0.49	0.63 (0.48-0.77)
Coagulopathy Positive (n) Negative (n)	31 03	13 09	1 (Ref.) 7.20 (1.66-30.76)	0.004	0.70 (0.55-0.83)
Cytokinaemia Positive (n) Negative (n)	14 20	05 17	1 (Ref.) 2.38 (0.71-7.97)	0.25	0.74 (0.49-0.91)
Liver inflammation Positive (n) Negative (n)	05 29	05 17	1 (Ref) 0.59 (0.15-2.32)	0.49	0.50 (0.19-0.81)

HICs: hyperinflammatory conditions, OR: Odds ratio, CI: confidence interval, 1 (Reference) N: total number of patients expired during the study period, and (n): the number of patients with (positive) or without (negative) specified conditions.

Regarding the significance of this study, the results presented above suggest that male COVID-19 patients developed more severe HICs, which greatly increased the risk for mortality compared to the female COVID-19 patients. In both univariate and bivariate analyses, macrophage activation, hematological dysfunction, coagulopathy and cytokinaemia were found to be strongly associated with severe male COVID-19 patients compared to the female COVID-19 patients. Webb et al. (2020) in a recent study proposed that the COVID-19 patients who develop two or more HICs may likely progress to mechanical ventilation and death [17]. In support of this, we found that nearly 85% male compared to 76% female COVID-19 patients, who expired had developed two or more HICs. Thus, our findings provide experimental support to various criteria developed by Webb et al. for predicting the severity and the risk for mortality in COVID-19 patients [17].

According to the epidemiological data, males are at a higher risk for COVID-19 transmission and mortality than females [10,11]. The reason for this is unclear; however, a strong lymphocyte and monocyte network may serve as a potential barrier, limiting virus transmission in females [4,17, 25,27,29,30,51,52]. In line with the reports, we observed that average/mean lymphocyte count was considerably greater in female COVID-19 patients than in male COVID-19 patients. About 82% male compared to 58% female severe COVID-19 patients developed lymphocytopenia (data not shown). These findings agree with earlier reports, which reported the development of severe lymphocytopenia in COVID-19 patients from China and other countries [2,4, 25,27,29,30,51,52]. While females, seems to have a stronger immune system that protects them from SARS-CoV-2 infection. Women over 50 have been shown to have greater lymphocyte counts and lower neutrophil to lymphocyte ratios than men of the same age, according to earlier research [53]. Furthermore, other studies have suggested that estrogens and progesterone in females, may help protect against SARS-CoV-2 infection [54].

**Figure 2.**
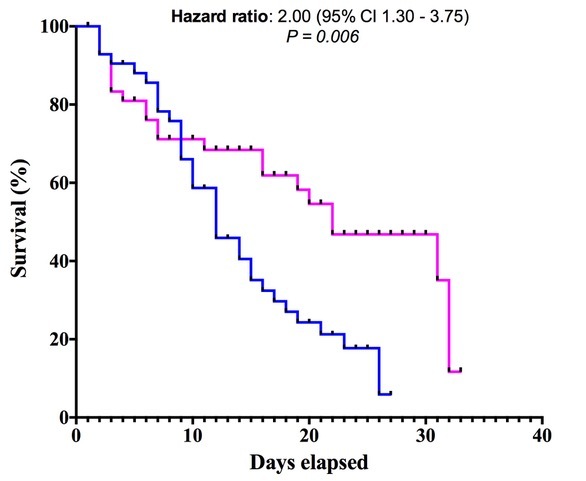
Survival curves of female and male COVID-19 patients (expired) generated using Kaplan-Meier method. Female COVID-19 patients (pink line) had significantly longer duration of survival (in-hospital stay) before death compared to the male COVID-19 patients (blue line). The hazard ratio was calculated using Logrank (Mantel-Cox test).

One of the crucial findings of this study was the prevalence of co-morbid factors including type-2 diabetes, hypertension, and renal disease (Table 1). In severe male COVID-19 patients, 24% had diabetes, 20% had renal disease, and 25% had hypertension. In total 69% severe male COVID-19 patients had pre-existing comorbidities. In severe female COVID-19 patients, 40% had diabetes, 18% renal disease, and 9% had hypertension. In total, 67% severe female COVID-19 patients had pre-existing comorbidities. Hypertension and renal disease were strongly associated with male COVID-19 patients (Table 1), whereas diabetes was a major risk factor female for developing COVID-19. Indeed, earlier studies have also reported that patients with various co-morbidities had significantly much higher risk for COVID-19 transmission and mortality [18-23].

Regarding therapeutic significance of the above findings, since the data and the supporting literature discussed above suggest that male COVID-19 patients developed relatively more severe HICs compared to the female COVID-19 patients, which could be the reason associated with relatively higher mortality rate in male COVID-19 patients. In particular, macrophage activation, coagulopathy, and cytokinaemia were strongly associated with mortality in male compared to the female COVID-19 patients. Since macrophage activation (hyperferritinaemia) increases the risk for hematological dysfunctions, coagulopathy and live inflammation, therapies aimed at reducing macrophage activation or hyperferritinaemia could be highly effective in reducing the severity and mortality associated with COVID-19. Foods or nutritional supplements, with low iron contents, and low content of vitamin C (which increases iron absorption) can significantly improve treatment outcome in COVID-19 patients.

## Supplementary Materials

The Supplementary data can be found online at: www.aginganddisease.org/EN/10.14336/AD.2022.0830.
