# Supplementary material for: Gender Differences Associated with Hyper-Inflammatory Conditions in COVID-19 Patients
Source: Aging Dis. 2023 Apr 1;14(2):299–308. doi: 10.14336/AD.2022.0830 (PMC10017144; doi:10.14336/AD.2022.0830)
Supplement: Supplementary file 1 — The Supplementary data can be found online at: www.aginganddisease.org/EN/10.14336/AD.2022.0830. [file AD-14-2-299-s.pdf]

SUPPLEMENTARY DATA

Gender Differences Associated with Hyper-Inflammatory Conditions in COVID-19 Patients

Fouzia Shoeb[1,2], Farzana Mahdi[2*], Imran Hussain[1,2]

© 2022. Shoeb F et al. Published online at http://www.aginganddisease.org/EN/10.14336/AD.2022.0830

SUPPLEMENTARY DATA

Supplementary Table 1. Association of various HICs with severe male and female COVID-19 patients (Bivariate analyses).

**HICs ↓**	**Male** N=132	**Female** N=78	**Bivariate** OR (95% CI)	***P***	**Sensitivity** (95 %CI)
MA+HD					
Positive (n, %)	52 (39.39)	16 (20.51)	1 (Ref)	0.01	0.76 (0.65- 0.86)
Negative (n, %)	80 (60.61)	62 (79.49)	2.50 (1.30-4.8)		
MA+CO					
Positive (n, %)	57 (42.18)	18 (23.08)	1 (Ref.)	0.005	0.76 (0.65-0.85)
Negative (n, %)	75 (56.82)	60 (76.92)	2.53 (1.35-4.75)		
MA+CY					
Positive (n, %)	20 (15.15)	04 (05.13)	1 (Ref.)	0.04	0.83 (0.63-0.95)
Negative (n, %)	112 (84.85)	74 (94.87)	3.30 (1.10-10.0)		
MA+LI					
Positive (n, %)	010 (07.58)	06 (07.69)	1 (Ref.)	0.79	0.63 (0.35-0.85)
Negative (n, %)	122 (92.42)	72 (92.31)	0.85 (0.29-2.44)		
HD+CO					
Positive (n, %)	73 (55.30)	28 (35.90)	1 (Ref)	0.01	0.72 (0.63-0.81)
Negative (n, %)	59 (44.70)	50 (64.10)	2.21 (1.24-3.93)		
HD+CY					
Positive (n, %)	22 (16.67)	09 (11.54)	1 (Ref)	0.42	0.71 (0.52-0.86)
Negative (n, %)	110 (83.33)	69 (88.46)	1.53 (0.67-3.52)		
HD+LI					
Positive (n, %)	08 (06.06)	04 (05.13)	1 (Ref)	1.00	0.67 (0.35-0.90)
Negative (n, %)	124 (93.94)	74 (94.87)	1.2 (0.35-4.10)		
CO+CY					
Positive (n, %)	28 (21.21)	10 (12.82)	1 (Ref)	0.14	0.74 (0.57-0.87)
Negative (n, %)	104 (78.79)	68 (87.18)	1.83 (0.84-4.01)		
CO+LI					
Positive (n, %)	12 (09.09)	08 (10.26)	1 (Ref)	0.81	0.60 (0.36-0.81)
Negative (n, %)	120 (90.91)	70 (89.74)	0.88 (0.34-2.25)		
CY+LI					
Positive (n, %)	08 (06.06)	03 (03.85)	1 (Ref)	0.75	0.73 (0.39-0.94)
Negative (n, %)	124 (93.94)	75 (96.15)	1.61 (0.42-6.27)		

HICs: hyperinflammatory conditions, OR: Odds ratio, CI: confidence interval, 1 (Reference)
N: total number of patients, and (n, %): the number, and percentage of patients with (positive) or without (negative) specified conditions, MA-macrophage activation, HD- hematological dysfunction, CO - coagulopathy, CY - cytokinaemia, LI - liver inflammation

SUPPLEMENTARY DATA

Supplementary Table 2. Association of various HICs with mortality in severe male and female COVID-19 patients (Bivariate analyses)

**HICs ↓**	**Male** N=34	**Female** N=22	**Bivariate** OR (95% CI)	***P***	**Sensitivity** (95 %CI)
MA+HD					
Positive (n, %)	19	08	1 (Ref)	0.18	0.70 (0.50-0.86)
Negative (n, %)	15	14	2.22 (0.74-6.67)		
MA+CO					
Positive (n, %)	11	06	1 (Ref.)	0.77	0.64 (0.38-0.86)
Negative (n, %)	23	16	1.28 (0.39-4.16)		
MA+CY					
Positive (n, %)	12	03	1 (Ref.)	0.21	0.79 (0.49-0.95)
Negative (n, %)	22	19	3.03 (0.74-12.46)		
MA+LI					
Positive (n, %)	03	03	1 (Ref.)	0.67	0.50 (0.12-0.89)
Negative (n, %)	31	19	0.61 (0.11-3.35)		
HD+CO					
Positive (n, %)	29	12	1 (Ref)	**0.01**	0.70 (0.54-0.84)
Negative (n, %)	05	10	4.83 (1.36-17.16)		
HD+CY					
Positive (n, %)	13	04	1 (Ref)	0.14	0.76 (0.50-0.93)
Negative (n, %)	21	18	2.79 (0.77-10.08)		
HD+LI					
Positive (n, %)	05	05	1 (Ref)	0.49	0.50 (0.19-0.81)
Negative (n, %)	29	17	0.59 (0.15-2.32)		
CO+CY					
Positive (n, %)	16	04	1 (Ref)	0.**05**	0.80 (0.56-0.94)
Negative (n, %)	18	18	4.00 (1.12-14.3)		
CO+LI					
Positive (n, %)	08	05	1 (Ref)	1.00	0.62 (0.32-0.86)
Negative (n, %)	26	17	1.05 (0.29-3.74)		
CY+LI					
Positive (n, %)	03	02	1 (Ref)	1.00	0.60 (0.15-0.95)
Negative (n, %)	31	20	1.07 (0.16-6.92)		

HICs: hyperinflammatory conditions, OR: Odds ratio, CI: confidence interval, 1 (Reference)
N: total number of patients expired during the study period, and (n): the number of patients with (positive) or without (negative) specified conditions, MA-macrophage activation, HD- hematological dysfunction, CO - coagulopathy, CY- cytokinaemia, LI - liver inflammation

© 2022. Shoeb F et al. Published online at http://www.aginganddisease.org/EN/10.14336/AD.2022.0830
